# A self-inducible heterologous protein expression system in *Escherichia coli*

**DOI:** 10.1038/srep33037

**Published:** 2016-09-09

**Authors:** L. Briand, G. Marcion, A. Kriznik, J. M. Heydel, Y. Artur, C. Garrido, R. Seigneuric, F. Neiers

**Affiliations:** 1 Centre des Sciences du Goût et de l’Alimentation, INRA, Université de Bourgogne Franche-Comté, F-21000 Dijon, France; 2 Université de Bourgogne Franche-Comté, Dijon, France; 3 INSERM, UMR 866, 7 blvd Jeanne d’Arc, 21000 Dijon, France; 4 UMR 7365 CNRS-Université de Lorraine IMoPA, 9 Avenue de la Forêt de Haye 54505 Vandoeuvre Les Nancy; 5 Anticancer Center Georges François Leclerc, Dijon, France

## Abstract

*Escherichia coli* is an important experimental, medical and industrial cell factory for recombinant protein production. The inducible lac promoter is one of the most commonly used promoters for heterologous protein expression in *E. coli*. Isopropyl-β-D-thiogalactoside (IPTG) is currently the most efficient molecular inducer for regulating this promoter’s transcriptional activity. However, limitations have been observed in large-scale and microplate production, including toxicity, cost and culture monitoring. Here, we report the novel SILEX (Self-InducibLe Expression) system, which is a convenient, cost-effective alternative that does not require cell density monitoring or IPTG induction. We demonstrate the broad utility of the presented self-inducible method for a panel of diverse proteins produced in large amounts. The SILEX system is compatible with all classical culture media and growth temperatures and allows protein expression modulation. Importantly, the SILEX system is proven to be efficient for protein expression screening on a microplate scale.

*Escherichia coli* is a versatile bacterium that has been recognized by drug regulatory authorities and grows rapidly to a high cell density on inexpensive carbon sources. *E. coli* is the host of choice for the first attempt at recombinant protein production, regardless of the original source^1^,^2^,^3^,^4^. One of the most commonly used *E. coli* expression systems relies on the inducible T7 RNA polymerase because this system obtains high yields of recombinant proteins^5^,^6^. The coding sequence of the T7 RNA polymerase is inserted into the bacterial chromosome under the control of the inducible lac UV5 operon and is transcribed by the endogenous *E. coli* polymerase. The lac repressor protein (LacI) regulates access to the T7 RNA polymerase coding sequence by binding to the lac UV5 operon. Protein expression induction is triggered by the addition of the inducer isopropyl-β-D-1-thiogalactopyranoside (IPTG), which is a structural non-metabolizable analogue of allolactose. The T7 RNA polymerase produced after induction specifically transcribes the coding sequence of the protein of interest that is inserted into the expression plasmid under the control of the T7 promoter^6^,^7^. Moreover, access to the plasmidic T7 promoter can be regulated by the lacI repressor when the T7 promoter is fused with the lac operator (T7lac promoter)^8^.

Several strategies have been developed over the past decades to improve the induction of expression in *E. coli.* IPTG is currently the most efficient method to induce promoter expression. However, this technique has the following limitations: (i) it requires cell culture monitoring to ensure that IPTG is added at the optimal cell density. Indeed, the induction point varies greatly from one recombinant protein to another, which makes the process difficult to automate, especially when several proteins are expressed in parallel (e.g., for a screen); (ii) it presents technical issues for small volumes; (iii) it is not compatible with industrial scale-up; (iv) it presents toxicity limitations (especially for human therapeutic protein production)^9^; and (v) it is not cost-effective.

The T7 system results in low recombinant protein expression during bacterial growth prior to induction. This phenomenon, which is commonly known as leaking, limits cell growth in cases of toxic recombinant protein production. Different approaches were designed to minimize or prevent this so-called leaking. Grossman *et al*. reported that the addition of 1% glucose to the medium led to the repression of the lac operon^10^. Another strategy consisted of inserting a plasmid encoding the T7 phage lysozyme into its namesake BL21(DE3)pLysS strain. The T7 lysozyme binds to the T7 RNA polymerase and inhibits transcription initiation, thereby lowering the expression of the genes under T7 promoter control and leading to a diminution of leaking^11^. However, leaking can also be an advantage for the expression of membrane proteins without IPTG induction, probably because slow expression does not saturate the Sec-translocon^12^.

To avoid IPTG, lactose can be used as an inducer during the transition from the exponential to the stationary phase^13^. Despite the absence of toxicity and its low cost, the use of lactose presents different limitations, including the development needed to identify the adequate induction conditions^14^. To partially solve these limitations, recent advances have focused on engineering a new strain that allows lactose induction^15^. This strain presents the advantage of avoiding the use of IPTG; however, the optical density (OD) must be monitored to ensure culture induction at the optimal cell density (generally corresponding to the middle of the log phase). The same authors have also developed a strain using galactose as an inducer with the same limitations^16^. Another strategy to solve the toxicity and cost limitations linked to IPTG induction is to engineer a new LacI that responds to non-metabolizable inducers such as gentiobiose, fucose or sucralose. However, this strategy does not solve the cell growth monitoring issue^17^. Recently, Studier proposed an auto-inducing medium that did not require IPTG induction^18^. This medium was calibrated by iteration to balance the glucose repression of the lac operon mentioned above and natural lactose induction under specific conditions.

To address these major limitations, we here report the SILEX (Self-InducibLe Expression) system based on a new strain that allows intrinsic efficient autoinduction without any changes to the culture medium. Our engineered BL21(DE3) strain (the SILEX system) contains only the SILEX plasmid encoding for the human heat shock protein 70 (hHsp70) and a second plasmid encoding for the protein of interest, thereby making SILEX the simplest inducible expression system to date.

## Results

### Human Hsp70 promotes autoinduction of its expression

**Figure 1:**
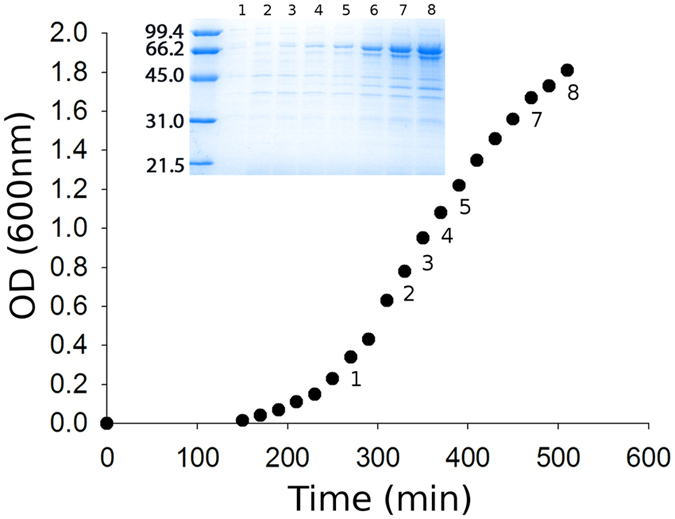
Autoinduction phenomenon during growth. Culture growth was monitored by optical density measurements at 600 nm (OD_60 nm_). Culture aliquots were analyzed during growth by SDS-PAGE to detect recombinant hHsp70 expression. A number on both the SDS-PAGE and the expression curve indicates which samples were chosen for the SDS-PAGE shown in the figure. The molecular weight markers are indicated on the left hand side of the gel in kDa (insert).

A pET28a plasmid containing the open reading frame (ORF) of the hHsp70 gene (SILEX plasmid) was used to transform the BL21(DE3) *E. coli* strain, leading to spontaneous autoinduction of the recombinant protein in the absence of IPTG induction (Fig. 1). Surprisingly, we observed that hHsp70 was expressed on a large scale and represented more than 50% of the overall bacterial proteins. Production primarily occurred during the log phase of cellular growth when the cell density reached approximately 9 × 10^8^ cells/mL. This spontaneous autoinduction phenomenon was not previously described for other recombinant proteins. As mentioned above, leaking can occur during heterologous protein expression, leading to the production of a low amount of the recombinant protein; however, leaking has never been demonstrated on such a large scale.

hHsp70 is a stress protein that presents an anti-aggregation function. hHsp70 interacts with many different protein partners to target misfolded proteins in human cells and to assess different physiological roles. We hypothesized that the observed autoinduction phenomena could be linked to any hHsp70 function with an *E. coli* human homologue partner or an interaction with a folded or misfolded protein.

### Deciphering the autoinduction mechanism

The strategy used to decipher the phenomenon was based on the identification of an interaction between the expressed recombinant hHsp70 and an unknown protein from the host organism (*E. coli*).

**Figure 2:**
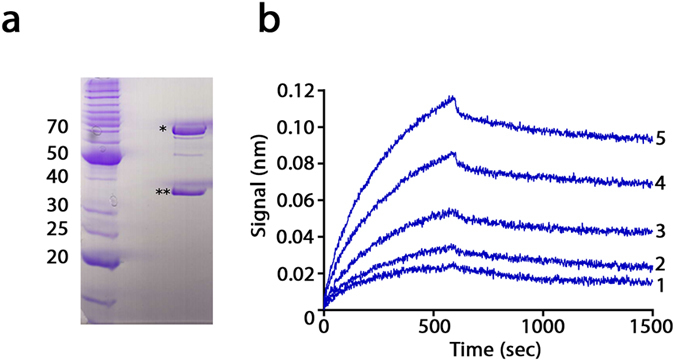
Isolation of the *E. coli* partner of hHsp70 and assessment of their interaction. (**a**) SDS-PAGE of the purified fraction containing the human Hsp70 (indicated by a single star) co-purified with a 35 kDa partner protein (indicated with a double star). (**b**) Sensorgrams were obtained by Bio-Layer Interferometry. Immobilized hHsp70 was deep in wells containing increasing *E. coli* GAPDH (1: 39.5 nM; 2: 59.3 nM; 3: 88.9 nM; 4: 133.3 nM and 5: 200 nM) A K_D_ value of 8.2 nM ± 0.2 nM (R^2^ of 0.986) was obtained with a 1:1 model of the OctetRED instrument software. The molecular weight markers are indicated on the left of the gels in kDa.

During hHsp70 purification (following our previously published protocol^19^), fractions contaminated with endogenous proteins were isolated during the first ion purification step. Interestingly, one *E. coli* protein was co-eluted with hHsp70 during the second size-exclusion chromatography step, suggesting that an interaction occurred between the two proteins (Fig. 2a). SDS-PAGE analysis showed that this endogenous protein migrated with an apparent molecular mass of 35 kDa. Peptide mass fingerprinting analysis revealed 27 different peptidic fragments covering 51% of the full-length protein that matched the glyceraldehyde 3-phosphate deshydrogenase (GAPDH) encoded by the *E. coli gapA* gene. The molecular mass of the *E. coli* GAPDH measured by SDS-PAGE was in agreement with the predicted value (35 kDa) deduced from the gene sequence. To confirm the interaction between hHsp70 and *E. coli* GAPDH, both proteins were heterologously expressed and purified. hHsp70 was produced using the previously described protocol^19^. *E. coli* GAPDH was expressed in *E. coli* and purified with a high degree of purity (>95%). The interaction between the two purified proteins was measured using a Bio-Layer interferometry (BLI) system (Octet Red, Pall Fortébio, Menlo Park, CA, USA). BLI is a label-free technology that is used to measure biomolecular interactions. The BLI analysis revealed a K_D_ value of 8.2 ± 0.2 nM between hHsp70 and *E. coli* GAPDH (Fig. 2b). This value demonstrates a high affinity between the two partners and excludes the possibility of a non-specific interaction. To explore this interaction, the affinity was measured between hHsp70 and human GAPDH. The BLI analysis demonstrated a stronger affinity with a K_D_ value of 1.3 ± 0.1 nM.

### Role of the plasmid leaking in the autoinduction phenomenon

We hypothesized that a low level of hHsp70 should accumulate during the first phase of bacterial growth due to the leakiness of the pET system. Due to their high affinity, hHsp70 can interact with endogenous GAPDH, leading indirectly and consequently to lacI removal and subsequently to a large amount of hHsp70 autoinduction.

To validate this hypothesis, different *E. coli* strains were tested for their ability to autoinduce hHsp70 production. BL21 Star (DE3) contains a rne131 gene mutation that results in a reduced level of the RNase E enzyme, which is involved in mRNA degradation. This strain presents higher mRNA stability and hence higher basal expression due to leaking. The highest expression of recombinant hHsp70 was observed with this strain, which was in line with our hypothesis. In parallel, the BL21(DE3)pLysS strain did not present any hHsp70 autoinduction, which was compatible with leaking being drastically reduced in this strain. The control strain DH5 alpha did not express the T7 polymerase, which excluded any hypothesis not linked to the T7 polymerase. The plasmid coding for hHsp70 was based on the pET28a plasmid (kanamycin-resistant); however, a pET21a plasmid (ampicillin-resistant) encoding hHsp70 also allowed autoinduction. Moreover, better autoinduction was observed for *E. coli* BL21 Star (DE3) with this new plasmid, which confirmed our hypothesis and excluded a link with the antibiotic resistance type.

### Efficient heterologous protein expression using the SILEX system

**Figure 3:**
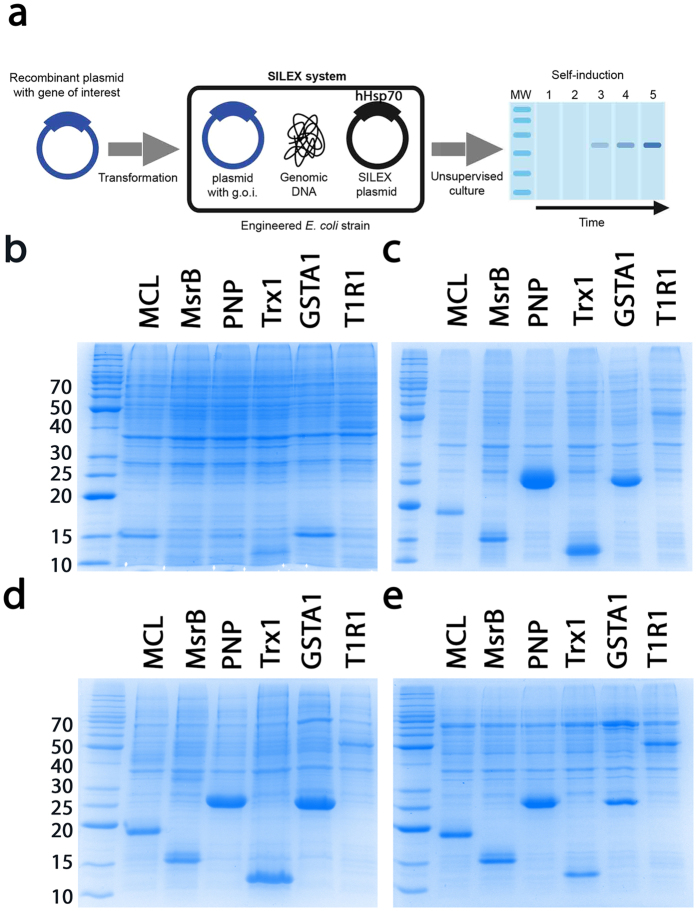
Principle of the SILEX system and application to 6 different recombinant proteins compared to the other primary expression system. (**a**) The use of SILEX can be divided in three steps, Step 1: As with other methods, a plasmid containing the coding sequence of the protein of interest (blue) is introduced (step 2) into host cells engineered from an *E. coli* strain containing a SILEX plasmid (black). Step 3: SDS-PAGE illustrating autoinduction in the SILEX system without the need to monitor the cell density or add a chemical inducer. Protein expression was monitor on SDS-PAGE after (**b**) BL21(DE3) growth without induction, (**c**) BL21(DE3) growth with IPTG induction, (**d**) BL21(DE3) growth in the ZYM auto-inducible medium, and (**e**) SILEX growth without any inducer addition. For each of the 6 proteins (*Richardella dulcifica* miraculin (MCL), *Xanthomonas campestris* methionine sulfoxide reductase B (MsrB), *E. coli* purine nucleoside phosphorylase (PNP), *E. coli* thioredoxin 1 (Trx1), *H. sapiens* glutathione transferase A1 (GSTA1) and *H. sapiens* taste receptor type 1 member 1 (T1R1), a cell aliquot was subjected to SDS-PAGE for expression analysis. The molecular weight markers are indicated on the left of the gels in kDa.

**Figure 4:**
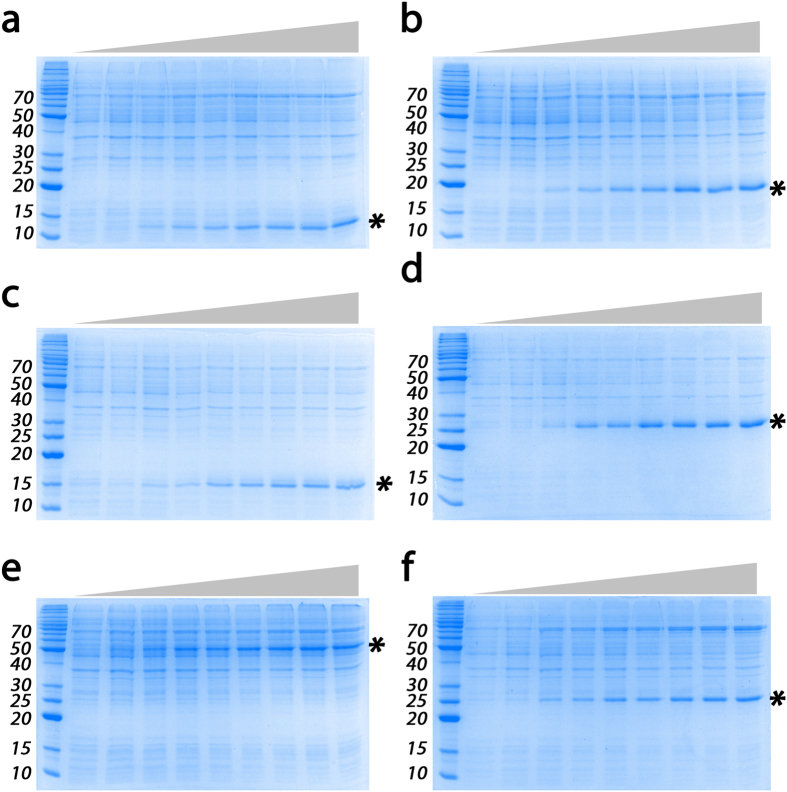
Autoinduction of recombinant proteins in SILEX. The expression levels of the 6 tested proteins are shown by SDS-PAGE in the panel: (**a**), *E. coli* Trx1, (**b**) *Richardella dulcifica* MCL, (**c**) *Xanthomonas campestris* MsrB, (**d**) *E. coli* PNP, (**e**) *H. sapiens* T1R1, and (**f**) *H. sapiens* GSTA1. The culture time is schematically represented on the top of the gel. The star indicates the recombinant protein. The recombinant protein is always autoinduced around an OD_600nm_ value of 1.0 as indicated in Table 1. The molecular weight markers are indicated on the left of the gels in kDa.

The autoinduction process reported for hHsp70 (i.e., SILEX plasmid) can be advantageously extended to express a protein of interest with the SILEX system by inserting the SILEX plasmid together with a second plasmid encoding the protein of interest into the bacterium. The second plasmid must have a different type of antibiotic resistance. SILEX systems were successfully tested with ampicillin or kanamycin-resistant plasmids, including a hHsp70-encoding pET plasmid with kanamycin or ampicillin resistance (pET28a or pET21a, respectively). To demonstrate the capabilities of the SILEX system, we chose a panel of 6 diverse proteins as follows: *Richardella dulcifica* miraculin (MCL), *Xanthomonas campestris* methionine sulfoxide reductase B (MsrB), *E. coli* purine nucleoside phosphorylase (PNP), *E. coli* thioredoxin 1 (Trx1), *H. sapiens* glutathione transferase A1 (GSTA1) and the N-terminal domain of *H. sapiens* taste receptor type 1 member 1 T1R1 (T1R1) (Supplemental Table 1). These 6 proteins span different (i) origins, including bacterial (MsrB, Trx1, and PNP), plant (MCL) and human (GSTA1 and T1R1), (ii) molecular masses (ranging from 14.0 kDa for Trx1 to 55.7 kDa for T1R1), (iii) functions (enzyme, receptor, ligand, and chaperone) and (iv) cellular localizations (e.g., cytoplasmic and plasma membrane). The T1R1 N-terminal domain, Trx1 and PNP were produced with a His_6_-tag. MCL were produced without the plant export signal. The plasmids used for protein expression were kanamycin-resistant with the exception of the plasmid encoding GSTA1, which carried an ampicillin resistance cassette. The 6 proteins produced by autoinduction with the SILEX system presented high levels of expression (Fig. 3) and were compared to the following situations where BL21(DE3) strain transformed with the different plasmids are: (i) induced with IPTG; (ii) induced using Studier autoinducing medium^18^; and (iii) not induced (control). A similar expression level was obtained in the 3 induced or autoinduced conditions (cultivated 24 hours after induction and started with a 1/50 preculture). As expected, the non-induced control did not express any protein. Interestingly, the SILEX system was also functional when the culture was started from a plate colony, thereby saving the time required for preculture. Interestingly, monitoring the protein expression level in the SILEX system allowed us to observe autoinduction of the 6 tested recombinant proteins during the culture growth period. This result strongly supports autoinduction against a continuous accumulation of the protein (Fig. 4).

**Figure 5:**
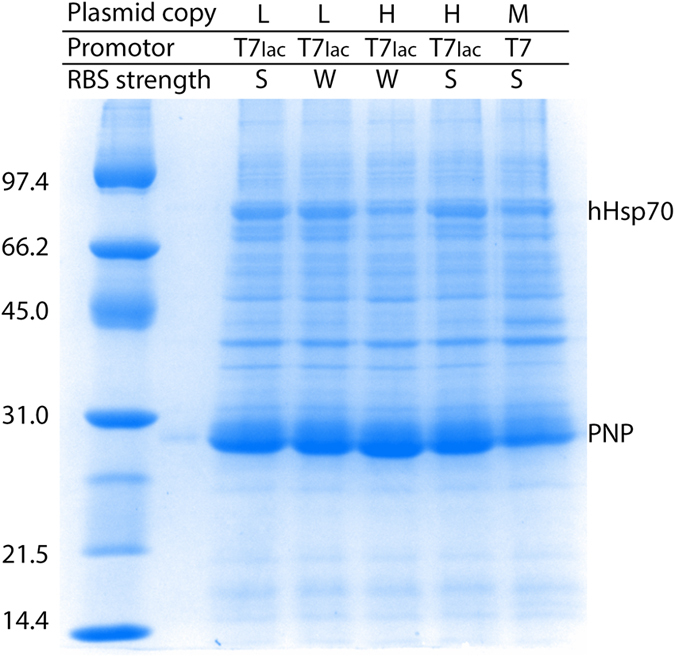
Autoinduction of PNP expression using different SILEX systems. The SILEX plasmid encoding hHsp70 was designed with different copy numbers (low or high), nature of the promoter (T7 or T7lac), and strength of the ribosome binding site (RBS) (strong or weak). For each resulting SILEX system, PNP expression was analyzed by SDS-PAGE. The bands corresponding to hHsp70 and PNP are indicated on the gel. The molecular weight markers are indicated on the left of the gels in kDa.

Curiously, expression with the SILEX system was significantly superior to hHsp70 expression for all tested proteins. In all cases, the plasmid containing the gene of interest was highly expressed even though the two plasmids (containing hHsp70 and the gene of interest) carried the same replication origin. Different parameters, such as the plasmid copy number (low or high), the nature of the promoter (T7 or T7lac), and the strength of the ribosome binding site (RBS) (strong or weak), were modulated on the hHsp70 expression plasmid to analyze the expression level of the protein of interest (PNP in Fig. 5) that was encoded by the second plasmid. The second plasmid used to express the PNP protein contained a replication origin encoding a medium copy number plasmid, a T7lac promoter and a strong RBS. First, PNP autoinduction was observed in all SILEX systems regardless of the tested feature of the hHsp70 expression plasmid. For all tested combinations, the PNP protein was always much more highly expressed than hHsp70 (Fig. 5). No obvious link could be established between the different protein expression levels, the plasmid copy number promoter type and the RBS strength, but these variations in expression could be used to tune the heterologous expression level. This result confirms the advantage of using SILEX to produce recombinant proteins, even though the higher expression level of the protein of interest compared to hHsp70 remains unexplained. This low hHsp70 expression is also an advantage for the purification of the protein of interest, indeed during the purification hHsp70 behaves as an endogenous *E. coli* contaminant.

### Modulation of the induction point

Glucose addition was previously demonstrated to indirectly modulate *lac* promoter expression. This phenomenon is known as carbohydrate-mediated inducer exclusion. The glucose PTS enzyme III is dephosphorylated by the entrance of glucose into the cell. Dephosphorylated PTS enzyme III binds to the lactose permease and inhibits lactose transport^20^. During the early stage of growth, 0.05% glucose was sufficient to block lactose. This concentration was tested as a supplement to the LB medium for all tested proteins. In other studies, lactose was demonstrated to be involved in the induction of lac promoter expression^10^,^13^. Lactose was tested at a concentration of 0.2% in the LB medium, which was the typical concentration described in the autoinducible medium developed by Studier^18^.

To measure the modulation of the autoinduction point, the expression of the proteins of interest (the 5 proteins encoded on the kanamycin-resistant pET plasmid) was tested in LB medium supplemented with or without glucose or lactose (LB, LB + 0.05% glucose, or LB + 0.2% lactose). The expression of each recombinant protein was monitored by SDS-PAGE and correlated to the OD_600 nm_ measurement (Table 1). The OD_60 nm_ before and after the first detection of heterologous protein is reported in Table 1 for each tested protein and each medium type. Autoinduction occurred at an average OD_60 nm_ of 1.0 ± 0.1 in LB medium at 37 °C for all tested proteins encoded by a similar plasmid type. The addition of glucose or lactose modulated the induction point, with a 0.05% final glucose concentration in the LB medium increasing the OD_60 nm_ to a level necessary to obtain autoinduction at an average of 1.5 ± 0.1 OD_60 nm._ The addition of lactose reduced the induction point to an average OD_60 nm_ of 0.7 ± 0.1.

### SILEX allows the generation and screening of thousands of expression conditions

Finding the optimal expression conditions for a given protein of interest requires searching a very large space of possible parameters, including the bacterial strains, culture media, growth temperatures and induction points. This process is currently unpredictable.

**Figure 6:**
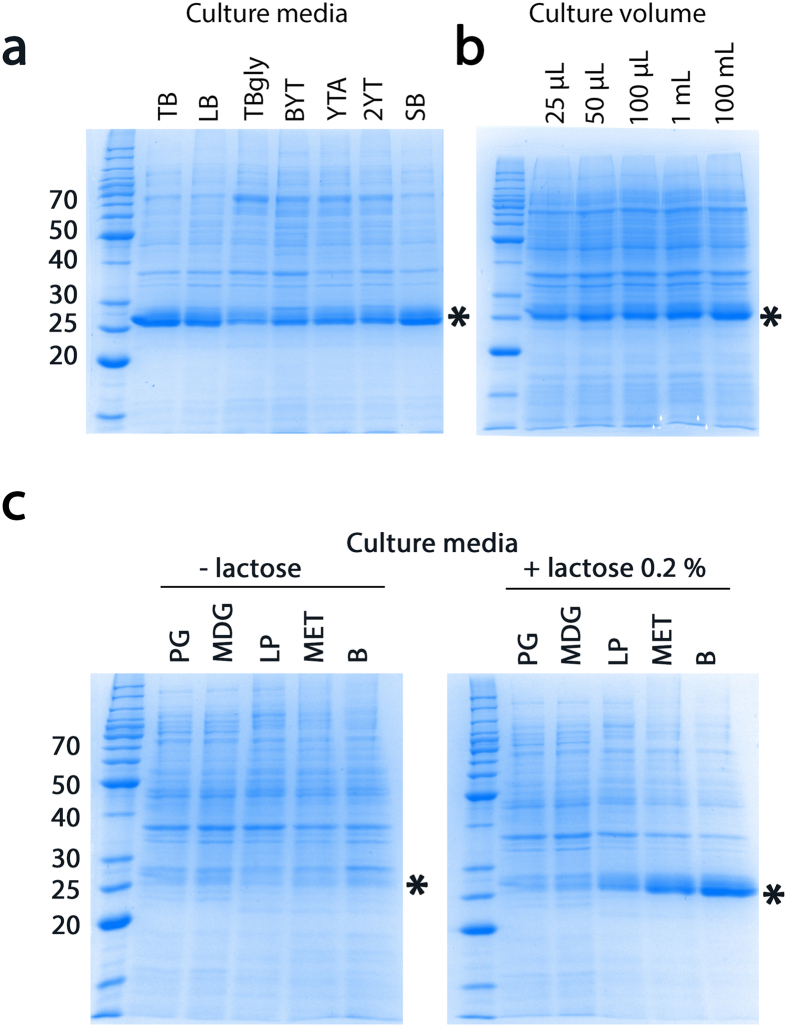
Autoinduction of PNP expression in the SILEX system using different media or culture volumes. For each medium or culture volume, PNP was expressed in the SILEX system. The expression was analyzed by SDS-PAGE and indicated by a star on the gel. The bands corresponding to hHsp70 and PNP are indicated on the gel. The molecular weight markers are indicated on the left of the gels in kDa.

Thousands of parameter combinations can be screened with the SILEX system because it (i) does not depend on a specific medium, (ii) is robust at various growth temperatures (20 °C, 25 °C, 30 °C or 37 °C), (iii) has a tunable induction point (based on the addition of either lactose or glucose), and (iv) works in a wide range of volumes from the μL to liter scale (see below and Fig. 6b).

We provide a sample of these capabilities with the PNP protein. The different SILEX versions presented in Fig. 5 do not express the same level of recombinant protein as shown for PNP expression (Fig. 5). Moreover, a large panel of medium types allows PNP autoinduction (Supplemental Table 2 and Fig. 6a). Interestingly, media containing an endogenous source of glucose prevented autoinduction. However, autoinduction could be restored with the addition of 0.2% lactose in 3 of the media types (Supplemental Table 2 and Fig. 6c,d). This result agreed with previous experiments that modulated the induction point.

The μL format (tested with 96-well microtiter plates) demonstrated the suitability of SILEX for the easy screening of several thousand possible different expression conditions. A low scale-up effect is required for the presented conditions tested for PNP expression (Fig. 6b). The scale-up effect can become more important depending on the conditions but generally allows better production at a higher volume.

## Discussion

The new system reported here named SILEX is the first system to allow recombinant protein overexpression using a lac inducible plasmid by autoinduction without any medium adaptation to date. Moreover, the system works on both small and large scales to allow easy expression screening.

SILEX relies on a metabolic modification driven by an interaction with the metabolic *E. coli* GAPDH enzyme. The existence of the *in vivo* interaction is directly supported by the finding that a fraction of the expressed hHsp70 was co-purified with *E. coli* GAPDH. Moreover, the BLI experiments allowed us to measure a strong affinity with a K_D_ value of 8.2 ± 0.2 nM between hHsp70 and *E. coli* GAPDH. The high sequence conservation of GAPDH due to the slow evolutionary rates^21^ explains the following findings: (i) the 64% identity measured between *H. sapiens* and *E. coli* GAPDH (Supplemental Fig. 1) and (ii) the conservation of the protein-protein interaction between the 2 species. Interestingly, the interaction measured between hHsp70 and *H. sapiens* GAPDH (K_D_ of 1.3 ± 0.1 nM) explained previous observations that suggested this interaction, including (i) co-immunoprecipitation experiments^22^ and (ii) the demonstration of specific recognition of a *H. sapiens* GAPDH peptide fragment by immobilized hHsp70^23^. These affinities also suggest the presence of a biological function in *H. sapiens* that is directly linked to this strong interaction, which may be investigated in the future. For example, the affinity of hHsp70 and human Hsp110, which is a well described physiological partner, is weaker, with a K_D_ value of 10 nM^24^.

**Figure 7:**
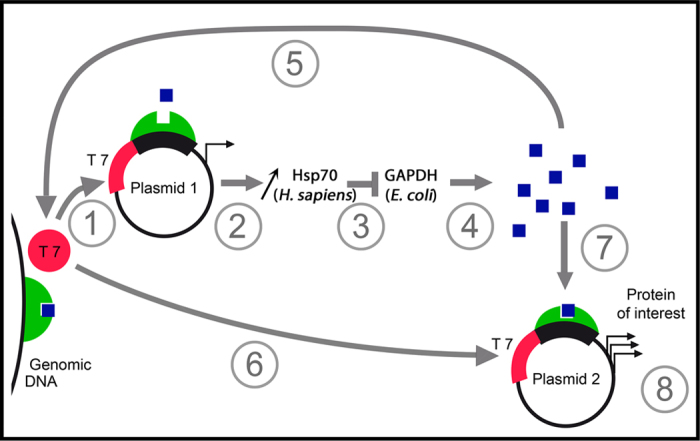
Schematic representation of the general mechanism of the autoinduction in SILEX. The figure summarizes the autoinduction mechanism. (1, 2) During the first stage of growth, a small quantity of heterologous hHsp70 is produced (coded by the plasmid 1). (3) hHsp70 interacts with endogenous *E. coli* GAPDH. (4) Progressively, the increase of *E. coli* GAPDH induces metabolic changes that most likely drive the strong induction. In this step, the lactose in the growth medium is necessary to accumulate the inducer represented by a blue square. (5) The induction of plasmid 1 leads to amplification of the phenomenon. Finally, the induction of the expression of plasmid 2 (6, 7) lead to high production of the protein of interest (8).

The conservation of the strong interaction between the two partners in *E. coli* allows the strong interaction between hHsp70 and *E. coli* GAPDH. This phenomenon is correlated with the low hHsp70 expression level due to leaking. The drastic reduction in leaking described for the BL21(DE3)pLysS strain led to the inhibition of autoinduction. In contrast, the BL21 Star (DE3) strain, which is characterized by increased leaking due to high mRNA stability, exhibits an increase in autoinduction. The direct consequences of the interaction between hHsp70 and endogenous *E. coli* GAPDH during leaking needs to be clarified in further studies. GAPDH is the 6^th^ enzyme in the glycolysis pathway. Disturbance of this enzyme can directly modify glycolysis and thus overall bacterial metabolism. The induction point occurs earlier during the lag phase when lactose is added to the initial medium, suggesting a role for lactose in autoinduction; this phenomenon was also observed with the panel of tested media (Fig. 6c). The presence of lactose in medium containing yeast extract was previously described and could explain the effectiveness of SILEX in these media^25^. A reasonable hypothesis may be that the interaction between *E. coli* GAPDH and hHsp70 decreases glucose metabolism, thereby favoring the lactose energy source; hence, lactose induction with either a low amount of lactose or residual glucose in the medium is favored. Carbohydrate-mediated inducer exclusion due to glucose is reinforced by the delay in autoinduction that was observed after adding glucose to the medium. This observation proves that competition occurs between lactose and glucose metabolism. Autoinduction was successfully tested for recombinant *E. coli* GAPDH overexpression. This result may seem counterintuitive at first. However, during the first stages of growth only a low amount of heterologous *E. coli* GAPDH is produced by leaking. This amount appears to be too low to disturb the interaction of hHsp70 with the endogenous *E. coli* GAPDH. To the best of our knowledge, the current working model we propose (although it is incomplete) is illustrated in Fig. 7.

Other groups have shown that autoinducible media may represent an interesting IPTG alternative. Studier’s group proposed tuning the medium composition by iteration, taking into account two main and opposite phenomena: glucose repression of the lac operon and the natural lactose induction that exists under specific conditions^18^ based on the preliminary observations of Grossman^10^. The main drawbacks of this approach are the cost and the few available types of complex media. Another approach consists of using other promoters induced by a metabolic state change during culture growth, such as oxygen, the pH level or the transition to depletion of a specific nutrient. Thus, culture growth also depends on a particular growth condition and/or culture medium^26^. The pharmaceutical company Novartis developed another autoinducible system that takes advantage of elements of the quorum sensing system of *Vibrio fischeri* to monitor cell density and produce commercial amounts of proteins (e.g., antigens) that can be used to prepare pharmaceutical compositions. However, this system requires a specific strain and plasmids^27^.

The SILEX system reported in this study is the first to allow overexpression of recombinant proteins using a lac inducible plasmid by autoinduction without any medium adaptation. SILEX works at different temperatures and on a panel of classical and diverse culture media (e.g., LB, TB, and 2YT) without any adaptation or with a simple lactose addition for other medium types (e.g., LP, MET, and B media). In contrast to existing approaches, our expression system proposes a major simplification and cost-effective alternative for protein production that does not require cell density monitoring or induction with expensive IPTG. Additionally, our yields of the tested proteins were equal to those obtained using classical IPTG induction. SILEX strains can be used for the production of a large variety of recombinant proteins, including proteins with human, bacterial or plant origins and proteins that are cytoplasmic or anchored to the membrane. One limitation of the SILEX system is the probable expression of toxic proteins due to the necessity for leaky expression. Beyond the proof-of-concept presented here, the SILEX system is extendable to other types of proteins and lac plasmids (e.g., T5 lac plasmids). Finally, SILEX combines the possibility of working in a 96-well microplate format with numerous testable conditions. The widespread use of SILEX in the future will provide a finer view of the system’s strengths and weaknesses compared to other protein expression systems in *E. coli*.

## Methods

### Media

The Luria Bertani broth medium (LB) used in this study was composed of 1.0% (w/v) tryptone, 0.5% (w/v) yeast extract and 0.5% (w/v) NaCl in distilled water. The pH was adjusted to 7.0 with a NaOH solution. Solid plates were obtained by adding 1.5% (w/v) agar. The antibiotic (45 mg/L of kanamycin or 100 mg/L of ampicillin (final concentration) or 25 mg/L of kanamycin and 50 mg/L of ampicillin added together) was added during plate preparation after solution cooling or to the LB medium prior to culture. The different growth media used in the study are described in Supplemental Table 2.

### Strains

The DH5 alpha *E. coli* strain (Invitrogen) was used to amplify plasmids and as a negative expression control. The BL21(DE3), BL21 Star (DE3) and BL21(DE3)pLysS strains used in the expression tests were purchased from Life Technologies (Carlsbad, CA, USA).

### Plasmids

A codon-optimized cDNA encoding hHsp70 in *E. coli* was synthesized by Geneart (Life Technologies, Carlsbad, CA, USA). The cDNA was subcloned into the pET21a^19^ or pET28a (this study) plasmid using the *NdeI* and *SacI* restriction sites for the insertion to generate the pET21hHsp70 and pET28hHsp70 plasmids, respectively. The same codon-optimized gene encoding hHsp70 was subcloned into the PD434-SR (low copy number p15a replication origin, strong RBS), PD434-WR (low copy number p15a replication origin, weak RBS), PD454-WR (high copy number pUC replication origin, weak RBS), and PJ414 plasmids (high copy number pUC replication origin, strong RBS) supplied by the DNA2.0 companies (Menlo Park, CA, USA) and the pET17b plasmid supplied by Life Technologies (Carlsbad, CA, USA).

The *Xanthomonas campestris* methionine sulfoxide reductase B (MsrB) open reading frame was subcloned from the pSKMsrBXc plasmid^28^ into the pET29b plasmid using the *NdeI* and *SacI* restriction sites including a stop codon before *SacI*. A synthetic gene containing the open reading frame of the *Richardella dulcifica* miraculine (MCL) was subcloned into pET28a using the *NcoI* and *XhoI* restriction sites including a stop codon before *XhoI* (this study). To obtain the pET28bTrx1 plasmid, the *trxA* open reading frame encoding *E. coli* thioredoxin 1 was amplified by PCR and inserted into the plasmid pET28b between the *NdeI* and *SacI* restriction sites (Trx1) (this study). pET28bPNP encoding *E. coli* purine nucleoside phosphorylase (PNP) and pET28-hT1R1-NTD encoding the *H. sapiens* T1R1 N-terminal domain (T1R1) were described in previous studies^29^,^30^,^31^. pET22GSTA1 encoding *H. sapiens* glutathione transferase A1 (GSTA1) was constructed in this study. The DNA sequence encoding *H. sapiens* GSTA1 was optimized for expression in *E. coli*, synthesized by DNA2.0 (Menlo Park, CA, USA), and subcloned into the pET22b vector between the *NdeI* and *SacI* restriction sites. The plasmids were transformed into BL21(DE3) competent cells with or without a SILEX plasmid encoding hHsp70. When not indicated, the SILEX system used to express the recombinant protein was constructed with the pET21hHsp70 or pET28hHsp70 plasmid with appropriate antibiotic resistance.

### Bacterial culture

The 25 μL, 50 μL, 100 μL, 1 mL and 100 mL cultures (in 96-well microplates, 24-well microplates, or 1, 10 and 500 mL Erlenmeyer flasks, respectively) were inoculated (1/50) from an overnight culture prepared with a loopful of one large isolated colony from a LB plate. When not indicated, the cultures were performed using LB medium. The shake flasks were cultured in an incubator shaker (INFORS AG, Bottmingen, Switzerland) with 25 mm offsets. The 96-well microplate and 24-well microplate were cultured in an incubator shaker (INFORS AG, Bottmingen, Switzerland) with 50 mm offsets. A box was used to avoid evaporation of the culture liquids in microplates (INFORS AG, Bottmingen, Switzerland). The rotation speed was 200 rpm. All results presented in the figures were obtained at 37 °C with 24 hours of growth after inoculation (1/50). The culture volume was 100 mL when not indicated. Expression using the ZYM autoinducible medium (ZYM-5052^18^) was obtained after 24 hours of growth. Expression using IPTG induction (1 mM final concentration, added between 0.5 and 0.6 OD_600 nm_) was also obtained after 24 hours of growth. The control culture without IPTG induction was obtained after 24 hours of growth.

### Cell growth measurement

Cell growth was determined by the optical density at 600 nm using a spectrophotometer (Cary 300 bio, VARIAN, Palo Alto, CA, USA). Suitable dilutions were generated in the corresponding LB medium to obtain an OD inferior to 1.5 for measurement.

### *E. coli* GAPDH plasmid construction, expression and purification

*Nde1* and *Sac1* restriction sites were inserted into the 5′ and 3′ ends, respectively, of the synthesized codon optimized *gapA* gene open reading frame (Geneart, Carlsbad, CA, USA). The digested sequences were ligated in a pET21a plasmid previously opened with the same restriction enzyme in the cloning cassette. The resulting expression vector pET21-*Ec*GAPDH encoded GAPDH. pET21-*Ec*GAPDH was transformed into *E. coli* BL21(DE3) (Novagen). A single colony from a LB–ampicillin agar plate (containing 100 mg/L ampicillin) was used to inoculate a 50 ml LB medium starter culture (containing 100 mg/L ampicillin) grown at 37 °C overnight. Then, 2 × 20 ml aliquots of the overnight culture were used to inoculate 2 × 1 L LB medium (containing 100 mg/L ampicillin). Expression was induced by the addition of 1 mM (final concentration) isopropyl-*d*-1-thiogalactopyranoside (IPTG) to cultures with an OD_600nm_ of 0.6. The cultures were maintained at 37 °C for 4 h following induction. Cells were harvested by centrifugation and resuspended in 20 mL of 50 mM Tris-HCl and 2 mM EDTA (pH 8) buffer containing 20 mM dithiothreitol (DTT). The resuspended cells were sonicated and centrifuged for 45 min at 20,000 × g. The supernatant was loaded onto a Q-Sepharose column coupled to a FPLC chromatography system (AKTA Purifer 10, GE). The protein was eluted with a salt gradient using a second buffer containing 50 mM Tris-HCl, 2 mM EDTA and 1 M KCl. The purity was checked using SDS-PAGE.

### Bio-Layer Interferometry (BLI)

BLI is an optical and label-free technique that is sensitive to an increase in the mass bound to the biosensor. In the first step, hHsp70 or human GAPDH was incubated in PBS buffer with biotin at a 1:3 molar ratio. Free biotin was removed using a desalting column (Pierce). Then, the biotinylated protein was immobilized onto streptavidin biosensor tips and dipped into wells containing *E. coli* GAPDH (39.5; 59.3; 88.9; 133.3 and 200 nM) or hHsp70 (2.05; 5.12; 12.8; 32 and 80 nM) in PBS buffer (hHsp70 and human GAPDH). After 600 s (association phase), the tips were dipped into wells containing 200 μL of PBS buffer for 900 s. The background was corrected, smoothed with the Savitzky-Golay algorithm and analyzed using the OctetRED instrument software (ForteBio Data Analysis version 7.1.). All sensograms were fitted with a 1:1 model that provided K_D_ values of 8.2 ± 0.2 nM (*E. coli* GAPDH with *h*Hsp70) and 1.3 ± 0.1 nM (*H. sapiens* GAPDH with *h*Hsp70) with R^2^ values of 0.986 and 0.995, respectively. The *k*_*on*_ of 2.93 ± 0.2 × 10^4 ^M^−1^.sec^−1^ and *k*_*off*_ of 2.40 ± 0.03 × 10^4 ^sec^−1^ were calculated for the interaction of *E. coli* GAPDH with hHsp70. The *k*_*on*_ of 7.24 ± 0.04 × 10^4 ^M^−1^.sec^−1^, and *k*_*off*_ of 9.3 ± 0.2 × 10^5 ^sec^−1^ were calculated for the interaction of *H. sapiens* GAPDH with *h*Hsp70.

## Additional Information

**How to cite this article**: Briand, L. *et al*. A self-inducible heterologous protein expression system in *Escherichia coli.* *Sci. Rep.* **6**, 33037; doi: 10.1038/srep33037 (2016).

## Supplementary Material

Supplementary Information

## Figures and Tables

**Table 1:** Modulation of the SILEX induction with lactose or glucose.

	LB (OD_60 nm_ before-after first recombinant protein detection)	LB + 0.05% glucose	LB + 0.2% lactose
MsrB	0.9–1.2 ± 0.1	1.5–1.6 ± 0.1	0.7–1.0 ± 0.1
Trx1	1.1–1.3 ± 0.1	1.6–1.8 ± 0.1	0.4–0.9 ± 0.1
PNP	1.0–1.1 ± 0.1	1.3–1.6 ± 0.1	0.6–1.0 ± 0.1
T1R1	1.0–1.3 ± 0.1	1.2–1.6 ± 0.1	0.5–0.8 ± 0.1
MCL	0.9–1.1 ± 0.1	1.6–1.8 ± 0.1	0.7–1.2 ± 0.1

To measure induction, a culture sample were analyzed by SDS-PAGE and the OD_60 nm_ was monitored every 15 minutes during log phase. For each recombinant protein, the OD_60 nm_ corresponding to the measurement point before and after first detection on SDS-PAGE was indicated in the table.
