# Supplementary material for: A self-inducible heterologous protein expression system in Escherichia coli
Source: Sci Rep. 2016 Sep 9;6:33037. doi: 10.1038/srep33037 (PMC5017159; doi:10.1038/srep33037)
Supplement: Supplementary Information [file srep33037-s1.doc]

**Supplementary Information**

A self-inducible heterologous protein expression system in *Escherichia coli*

Briand L, Marcion G, Kriznik A, Heydel JM, Artur Y, Garrido C, Seigneuric R, Neiers F

*H. sapiens GAPDH* MGKVKVGVNG FGRIGRLVTR AAFNSGKVDI VAINDPFIDL NYMVYMFQYD STHGKFHGTV KAENGKLVIN GNPITIFQER

*E. coli GAPDH* -MTIKVGING FGRIGRIVFR AAQKRSDIEI VAIND-LLDA DYMAYMLKYD STHGRFDGTV EVKDGHLIVN GKKIRVTAER

*H. sapiens GAPDH* DPSKIKWGDA GAEYVVESTG VFTTMEKAGA HLQGGAKRVI ISAPSAD-AP MFVMGVNHEK YDNSLKIISN ASCTTNCLAP

*E. coli GAPDH* DPANLKWDEV GVDVVAEATG LFLTDETARK HITAGAKKVV MTGPSKDNTP MFVKGANFDK YAG-QDIVSN ASCTTNCLAP

*H. sapiens GAPDH* LAKVIHDNFG IVEGLMTTVH AITATQKTVD GPSGKLWRDG RGALQNIIPA STGAAKAVGK VIPELNGKLT GMAFRVPTAN

*E. coli GAPDH* LAKVINDNFG IIEGLMTTVH ATTATQKTVD GPSHKDWRGG RGASQNIIPS STGAAKAVGK VLPELNGKLT GMAFRVPTPN

*H. sapiens GAPDH* VSVVDLTCRL EKPAKYDDIK KVVKQASEGP LKGILGYTEH QVVSSDFNSD THSSTFDAGA GIALNDHFVK LISWYDNEFG

*E. coli GAPDH* VSVVDLTVRL EKAATYEQIK AAVKAAAEGE MKGVLGYTED DVVSTDFNGE VCTSVFDAKA GIALNDNFVK LVSWYDNETG

*H. sapiens GAPDH* YSNRVVDLMA HMASKE

*E. coli GAPDH* YSNKVLDLIA HISK--

**Supplemental figure 1.** *Sequences alignment for the E. coli and H. sapiens GAPDHs.*

The sequences were aligned using the BioEdit software (version 7). The yellow highlight indicates similar residues. The two sequences present 64% identity.

| **Protein** | **Name** | **Uniprot ID**  **(NBCI gene ID)** | **MW**  **kDa** | **Main function(s)** | **Main location** | **Organism** |
| --- | --- | --- | --- | --- | --- | --- |
| Trx1 | thioredoxin 1  (his_6_-tag**, thrombin site)** | P0AA25  (948289) | 14.0 | Redox enzyme | cytoplasm | *Escherichia coli* |
| MsrB | methionine sulfoxide reductase B | Q8P4Q6  (1000226) | 16.8 | Redox enzyme | cytoplasm | *Xanthomonas campestris* |
| MCL | miraculin  (without signal peptide: 29 first amino acids) | P13087  (6166552) | 21.3 | Taste modifier | intracellular space | *Richardella dulcifica* |
| PNP | purine nucleoside phosphorylase  (his_6_-tag**, thrombin site)** | P0ABP8  (945654) | 28.1 | Purine metabolism enzyme | cytoplasm | *Escherichia coli* |
| GSTA1 | glutathione transferase | P08263  (2938) | 25.6 | Transferase | cytoplasm | *Homo sapiens* |
| **T1R1** | **taste receptor type 1 member 1 N-terminal domain (**his_6_-tag**, thrombin site, Phe21 to Ser 495)** | **Q7RTX1**  **(80835)** | **55.7** | **Domain of the sweet taste receptor** | **cell surface** | **Homo sapiens** |
| **Hsp70** | **inducible heat shock protein 70** | **P54652**  **(3306)** | **70.0** | **Enzyme, chaperone** | **cytoplasm** | **Homo sapiens** |

**Supplemental table 1: *Characteristics of*** *the 6 tested proteins and the hHsp70 used in the SILEX system.*

MW: Molecular weight.

|  | **TB** | **LB** | **TB + glycerol** | **BYT** | **YTA** | **2YT** | **SB** | **PG** | **MDG** | **LP** | **Metabolic** | **B** |
| --- | --- | --- | --- | --- | --- | --- | --- | --- | --- | --- | --- | --- |
| Na_2_HPO_4_ (mM) |  |  |  | 63.4 | 31.7 |  |  | 50 | 25 | 84.5 | 63 |  |
| NaH_2_PO_4_-H_2_O (mM) |  |  |  |  |  |  |  |  |  |  |  | 26 |
| KH_2_PO_4_ (mM) | 16.2 |  | 16.2 | 7.3 | 3.7 |  |  | 50 | 25 | 44 | 55 |  |
| K_2_HPO_4_ (mM) | 54 |  | 54 |  |  |  |  |  |  |  | 57 | 72.3 |
| NaCl (mM) |  | 171.2 |  |  |  | 85.6 | 85.6 |  |  | 8.6 |  |  |
| NH_4_Cl (mM) |  |  |  |  |  |  |  |  | 50 | 18.7 | 18.7 | 9 |
| FeCl_2_ (µM) |  |  |  |  |  |  |  |  |  | 10 |  |  |
| MgSO_4_ (mM) |  |  |  |  |  |  |  | 2 | 2 | 4 |  | 3 |
| CaCl_2_ (µM) |  |  |  |  |  |  |  |  |  | 100 |  |  |
| H_3_BO_3_ (µM) |  |  |  |  |  |  |  |  |  | 0.4 |  |  |
| CoCl_3_ (µM) |  |  |  |  |  |  |  |  |  | 0.03 |  |  |
| MnCl_2_ (µM) |  |  |  |  |  |  |  |  |  | 0.08 |  |  |
| ZnSO_4_ (µM) |  |  |  |  |  |  |  |  |  | 0.01 |  |  |
| Na_2_SO_4_ (mM) |  |  |  |  |  |  |  |  | 5 |  |  | 14 |
| Aspartate (%) |  |  |  |  |  |  |  |  | 0.25 |  |  |  |
| (NH_4_)_2_SO_4_ (mM) |  |  |  |  | 249.7 |  |  | 25 |  |  |  | 46 |
| (NH_4_)_2_H-Citrate (mM) |  |  |  |  |  |  |  |  |  |  |  | 4.4 |
| Thiamine hydrochloride (mM) |  |  |  |  |  |  |  |  |  |  | 0.089 | 0.3 |
| Tryptone (%) | 1.2 | 1 | 1.2 | 1 | 1 | 1.6 | 3.5 |  |  |  |  | 0.18 |
| Yeast extract (%) | 2.4 | 0.5 | 2.4 | 0.5 | 1 | 1 | 2 |  |  |  | 0.0001 | 0.18 |
| K_2_SO_4_ (mM) |  |  |  |  |  |  |  |  |  |  | 13.8 |  |
| FeSO_4_-(_7_H_2_O) (µM) |  |  |  |  |  |  |  |  |  |  | 220 |  |
| CaCl_2_-(_2_H_2_O) (µM) |  |  |  |  |  |  |  |  |  |  | 410 |  |
| MnCl_2_-(_4_H_2_O) (µM) |  |  |  |  |  |  |  |  |  |  | 60 |  |
| CoCl_2_-(_6_H_2_O) (µM) |  |  |  |  |  |  |  |  |  |  | 34 |  |
| ZnSO_4_-(_7_H_2_O) (µM) |  |  |  |  |  |  |  |  |  |  | 24 |  |
| CuCl_2_-(_2_H_2_O) (µM) |  |  |  |  |  |  |  |  |  |  | 18 |  |
| H_3_BO_3_(µM) |  |  |  |  |  |  |  |  |  |  | 3.2 |  |
| (NH_4_)_6_Mo_7_O_24_-(4H_2_O) (µM) |  |  |  |  |  |  |  |  |  | 0.003 | 1.9 |  |
| EDTA (µM) |  |  |  |  |  |  |  |  |  |  | 120 |  |
| MgCl_2_ (mM) |  |  |  |  |  |  |  |  |  |  | 10 |  |
| Glucose (%) |  |  |  |  |  |  |  | 0.5 | 0.5 | 2 | 0.2 | 0.1 |
| Glycerol (%) |  |  | 1 |  |  |  |  |  |  |  |  |  |

**Supplemental table 2:** *Culture media compositions.*
